# Parenteral Transmission of the Novel Human Parvovirus PARV4

**DOI:** 10.3201/eid1309.070428

**Published:** 2007-09

**Authors:** Peter Simmonds, Ashleigh Manning, Rachel Kenneil, Frances W. Carnie, Jeanne E. Bell

**Affiliations:** * University of Edinburgh, Edinburgh, Scotland, United Kingdom; † Western General Hospital, Edinburgh, Scotland, United Kingdom

**Keywords:** Parvovirus, PARV4, B19, parenteral transmission, injection drug use, persistence, HIV, AIDS, hemophilia, hepatitis C virus, dispatch

## Abstract

Transmission routes of PARV4, a newly discovered human parvovirus, were investigated by determining frequencies of persistent infections using autopsy samples from different risk groups. Predominantly parenteral routes of transmission were demonstrated by infection restricted to injection drug users and persons with hemophilia and absence of infection in homosexual men with AIDS and low-risk controls.

The novel human parvovirus PARV4 is a recently discovered member of the family *Parvoviridae* (*1*). PARV4 was originally cloned from a sample obtained from a person at risk for HIV infection through injection drug use or sexual contact who was enrolled in the San Francisco-based Option Project cohort. Since the original report, PARV4 infections have been detected in samples of pooled plasma from a variety of manufacturers (*2**,**3*), and at low frequency in pooled and individual plasma samples from US blood donors (<2%) (*3*). A higher frequency of PARV4 viremia found among other persons enrolled in the Option Project cohort (6%) (*3*) and detection of PARV4 in autopsy blood from a person with a history of injection drug use (*4*) are indicators of a potential association between PARV4 infection and high-risk behavior for HIV infection.

Despite the acute, resolving nature of many parvovirus infections, it is now well established that many members of the family *Parvoviridae*, such as the human erythrovirus B19, can establish lifelong persistence with restricted replication and absence or rarity of detectable long-term viremia (*5**–**7*). Using a highly sensitive nested PCR, we recently investigated a range of autopsy tissues taken from high-risk persons (HIV-infected with histories of injection drug use or male homosexual contact) and low-risk persons (uninfected with HIV and hepatitis C virus [HCV]) who did not have a history of parenteral exposure or contact with multiple sexual partners. We checked for evidence of similar persistence of PARV4 (*8*). Remarkably, bone marrow, lymphoid tissue, or both from 17 of 24 study subjects in the high-risk group were positive for PARV4, but both sample types were uniformly negative in low-risk controls. These findings not only confirm the ability of PARV4 to establish persistent infections in humans, but also the existence of shared risk factors with HIV for transmission.

## The Study

We tested well-defined risk groups for HIV and parenterally transmitted infections to more precisely determine the transmission route of PARV4. Autopsy tissue samples used in this project were obtained from the Edinburgh Medical Research Council (MRC) HIV Brain and Tissue Bank at the Western General Hospital, Edinburgh. Consent for use of postmortem tissue was obtained from the Lothian Research Ethics Committee (LREC2002/4/36). Study subjects were divided into 4 groups of approximately equal size (n = 11–13, Table 1): 1) injection drug users (IDUs) without HIV infection (all positive for antibodies to HCV when testing was available); 2) Men who had sex with men (MSM) with AIDS without a history of parenteral exposure (all negative for antibodies to HCV when testing was available); 3) IDUs infected with HIV with AIDS-defining illnesses; and 4) IDUs infected with HIV who died of other causes while presymptomatic.

**Table 1:** Detection of parvovirus B19 and PARV4 in study groups*

Participant category (no.)	M/F	Age at death, y (range)†	Year of death (range)†	Mean CD4/μL (range)†	AIDS?	B19 positive, no. (%)	PARV4 positive, no. (%)
HIV+ IDU, AIDS‡ (13)	10/3	35 (2–48)	1995 (1991–1998)	44 (1–137)	Y	6 (46)	11 (85)
HIV+ IDU, pre-AIDS‡ (11)	7/4	33 (29–40)	1996 (1992–1998)	268 (167–496)	N	6 (55)	6 (55)
HIV+ MSM (13)	13/0	39 (28–49)	1993 (1990–1996)	25 (1–160)	Y	7 (54)	0
HIV– IDU (12)	10/2	35 (24–49)	1999 (1992–2005)	ND	NA	8 (67)	1 (8)
Hemophilia (2)	2/0	22, 26	1994, 1995	0	Y	0	1 (50)
Low-risk control§ (8)	3/5	54 (28–89)	All 2005	ND	NA	8 (100)	0

*IDU, injection drug user; M, male; F, female; Y, yes; N, no; MSM, men who had sex with men; ND, not done; NA, not applicable. †Values are means (ranges) or individual values for those with hemophilia. ‡These study groups overlap with the previously analyzed HIV-positive group (*8*), restricted to those in whom parenteral risk factors for infection have been identified. §Previously described in (*8*).

We also tested samples from 2 persons with hemophilia treated with nonvirally inactivated factor VIII concentrates from the late 1970s onward, both of whom became infected with HIV and HCV. Study subjects showed similar demographic characteristics, with similar age ranges and dates of death largely restricted to the 1990s (Table 1). The IDUs, MSM, and patients with hemophilia with AIDS showed similar mean CD4 counts before death, indicating profound immunosuppression. Samples of lymphoid tissue (lymph node or spleen) and bone marrow were assayed for parvovirus B19 and PARV4 DNA sequences by nested PCR as described (*8*). In all samples, >0.5 μg of genomic DNA was tested, which provided a test sensitivity of ≈6 copies of target sequence/10^6^ cells. Detection of both parvovirus B19 and PARV4 sequences was highly reproducible between the 2 tissues analyzed (Table 2), which enabled generally unambiguous categorization of study subjects into infected and uninfected categories. Persons in whom B19 or PARV4 was detected in 1 of the 2 tissues were considered infected, although the same conclusions for risk group associations would have been reached if the 5 persons with discrepant results had been excluded from analysis or considered uninfected (data not shown).

**Table 2:** Concordance of parvovirus B19 and PARV4 detection between bone marrow and lymphoid tissue*

Virus detected and risk group	Lymphoid tissue	
	Positive	Negative
B19 (all risk groups)		
Bone marrow		
Positive	27	1
Negative	3	29
PARV4 (IDUs and persons with hemophilia)		
Bone marrow		
Positive	14	1
Negative	2	21

*IDUs, injection drug users.

Parvovirus B19 infection frequency increased with age of the patients and corresponded closely to frequencies of B19 seropositivity in the general population in the United Kingdom recorded previously for different age ranges (*9*). Infections were absent in 2 young patients with hemophilia (22 and 26 years of age at death), ranged from 46% to 67% in IDUs and MSM (mean ages 33–39 years), and were found in all 8 low-risk controls (mean age 54 years). These findings provide further evidence for high frequencies of or potentially universal persistence of infection with B19 in those exposed (*5**–**7*). In contrast, infections with PARV4 were restricted to those with a history of parenteral exposure (IDUs and patients with hemophilia). Frequencies of infection ranged from 8.3% (1/12) of the HIV-negative IDUs to 55% and 85%, respectively, in HIV-infected IDUs before and after AIDS developed. Similarly, 1 of the 2 patients with hemophilia was positive for PARV4. No PARV4 infections were found in the MSM group, despite profound immunosuppression associated with AIDS and histories of frequent past exposure to sexually transmitted infections, such as HIV.

## Conclusions

The absence of detectable PARV4 in the MSM group demonstrates that PARV4 infections are not specifically associated with HIV co-infection. Instead, its specific risk group association with injection drug use and hemophilia (and absence in MSM and low-risk controls) provides evidence for a predominantly or exclusively parenteral route of transmission. The higher frequency of PARV4 detection in HIV-positive persons may be an indirect reflection of the greater degree of parenteral exposure among IDUs who become infected with HIV. In Edinburgh, HIV infections are much less prevalent in the IDU population than are HCV infections, because the transmission of HIV is less efficient through the bloodborne route (*10*). The higher frequency of PARV4 infection in the IDU-AIDS group compared with the frequency in the pre-AIDS group may also have originated through differential parenteral exposure; AIDS is more likely to be diagnosed among those exposed early to HIV in their period of injection.

It could be argued that the higher frequency of PARV4 infection in HIV-infected IDUs and persons with hemophilia may be the result of greater ease of detection in immunosuppressed persons. PARV4 infections may be widespread like B19 infections but may only persist in detectable amounts in persons whose compromised immune system allows viral reactivation. Although this hypothesis was difficult to discount in our original study (*8*), our new observation of an absence of PARV4 infection in MSM with AIDS removes the proposed link between immunosuppression and PARV4 detection.

The findings of our study provide evidence that PARV4 is primarily or exclusively transmitted through parenteral routes, a marked contrast to predominantly respiratory routes of transmission of parvoviruses in other genera, including B19 and human bocavirus (*11*). Although no information was provided on the specific risk factors for persons at high risk who were enrolled in the Options Project cohort (i.e., whether an IDU or MSM), the finding of a higher prevalence of viremia in this group compared with blood donor controls (*3*) is also consistent with evidence obtained in the current study for a predominantly parenteral route of transmission.

Previous observations of the remarkable sequence homogeneity of PARV4 nucleotide sequences between variants detected in the United Kingdom and in the United States (*3**,**8*) indicate the recent origin and spread of this virus in this specific risk group. Although we currently understand little about its pathogenicity or the clinical outcome of infection, PARV4 infection represents a potential newly emerging, additional bloodborne virus in IDUs. Given the resistance of parvoviruses to viral inactivation procedures, recipients of a wide range of plasma-derived therapeutics may also be at risk for PARV4 infection.
